# Corticoid injection as a predictive factor of results of carpal tunnel release

**DOI:** 10.1590/1413-78522015230200943

**Published:** 2015

**Authors:** Giselly Veríssimo de Miranda, Carlos Henrique Fernandes, Jorge Raduan, Lia Miyamoto Meirelles, João Baptista Gomes dos Santos, Flávio Faloppa

**Affiliations:** 1 Universidade Federal de São Paulo, Escola Paulista de Medicina, São Paulo, SP, Brazil, 1. Escola Paulista de Medicina da Universidade Federal de São Paulo (EPM-UNIFESP), São Paulo, SP, Brazil

**Keywords:** Adrenal cortex hormones, Prognosis

## Abstract

**OBJECTIVE:**

To evaluate whether the symptoms relief after local corticoid injection correlate with better results of surgical treatment in carpal tunnel syndrome.

**METHODS:**

Between February 2011 and June 2013, 100 wrists of 88 patients were included in the study. All patients were subjected to corticoid injections in the carpal tunnel and evaluated before and after infiltration and surgery. The following parameters were evaluated: visual analog scale (VAS) for pain, Boston questionnaire, sensitivity and strength.

**RESULTS:**

Only 28 out of 100 wrists subjected to injection were symptom-free after six months follow up. Sixty out of the 72 patients who did not present relief or relapse symptoms were treated surgically. Surgical results were better regarding VAS, Boston questionnaire and sensitivity in a specific group of patients, which had a longer relief of symptoms after the corticoid injection, with statistical significance.

**CONCLUSION:**

Longer relief of symptoms after corticoid injection correlated with better results of surgical treatment.

**Level of Evidence II, Prognostic Study.:**

## INTRODUCTION

Carpal tunnel syndrome (CTS) is the most common compressive neuropathy of the upper limb. It is defined as a compressive neuropathy of the median nerve in the wrist causing symptoms. In the United States, the incidence is one to three cases per thousand people per year and a prevalence of 50 cases per thousand people per year.^1^

The diagnosis of carpal tunnel syndrome is controversial, based on the clinical condition and the results of electromyography. The lack of accuracy in the diagnosis has been identified as one of the most common causes of treatment failure for carpal tunnel syndrome.^2^

For the diagnosis, clinical criteria are described in the literature, such as paresthesia in the median nerve territory, nighttime paresthesia, thenar atrophy, Tinel test, Phalen test, and decreased two point discrimination test (greater than 6 mm).^2^

Treatment for CTS may be surgical or non-surgical. There are several options in both treatment modalities. Among the non-surgical treatment options there are local corticosteroids injection, oral corticosteroids, night orthesis and physical therapy. Symptoms improvement after 12 months of non-surgical treatment with these methods is only 11%.^3^ The surgical treatment can be performed by open or endoscopic techniques. There is evidence that surgical treatment is better than non-surgical treatment, especially in severe cases. There is no evidence of superiority regarding the surgical technique used.^4^^,^ 5

Patient satisfaction rates with improvement of symptoms after surgical treatment range from 54 to 94%.^6^^,^ 7 Because of this variation, several studies have attempted to identify some predictive factor for the results of surgical treatment of carpal tunnel syndrome. The results were correlated with electroneuromiography tests and magnetic resonance imaging. We found no correlation as a reliable predictive factor.^8^^,^ 9

Retrospective studies have shown a positive correlation between symptom improvement after intracanal corticosteroid injection and symptom improvement after surgery, however, no prospective studies were found in the literature correlating these data.^7^

Patients with CTS that respond well to corticosteroids intracanal infiltration have better postoperative results.

The aim of this study was to evaluate whether the improvement in the symptoms of carpal tunnel syndrome after local injection of corticosteroids is a positive predictor of response to surgical treatment in cases of recurrence of symptoms after infiltration.

## MATERIALS AND METHODS

The study was conducted prospectively from March 2011 to June 2013 with the approval of the Research Ethics Committee of the institution.

A total of 88 patients diagnosed with carpal tunnel syndrome were evaluated, totaling 100 wrists.

Of the 88 patients, 83 were female and 5 male. The mean age was 50.5 years old, standard deviation 8.5 years old.

The infiltrated side was the dominant hand side in 53 patients, 60% of cases.

Among female patients, 54 were in perimenopause or postmenopause.

The mean duration of symptoms was 5.4 years, with a standard deviation of 4 years. Paresthesia was the most common symptom, present in 92 wrists. Only night paresthesia was reported in 25 wrists, while on the other 67, patients complained of paresthesia both at night and daytime.

On physical examination, Durkan and Phalen tests were positive in 89 and 85 wrists, respectively and the Tinel test was positive in only 64 wrists.

Only 18 wrists had not been subjected to other forms of non-surgical treatment. The other 82 reported use of orthosis, physical therapy and analgesics and anti-inflammatory drugs, without improvement.

To diagnose the presence CTS it was required three or more diagnostic criteria according to the parameters of the American Academy of Orthopedic Surgeons, which are:^1^^,^ 2

1. Paresthesia in the median nerve territory
2. Night paresthesia
3. Thenar atrophy
4. Positive Tinel test
5. Positive Phalen test
6. Decreased sensitivity.

Exclusion criteria were: diagnosis of diabetes, hypothyroidism or fibromyalgia, previous surgical treatment or local corticosteroids infiltration.

Eligible patients were informed about the study and signed the Free and Informed Consent Form in order to participate.

After signing the consent form, the patients were included in the study.

Patients underwent assessment by an independent examiner. The chronogram included four assessments before infiltration at one, three and six months after infiltration. The parameters evaluated were:

• Boston-Levine questionnaire validated for the Portuguese language,^10^
• Visual analogue pain scale,^11^
• Sensitivity with nylon monofilament (SORRI^(r)^ esthesiometer),^12^
• Hand grip strength
• Digital pinch strength
• Pulp to pulp pinch,
• Tridigital pinch,
• Lateral pinch, and
• Complications of the method

The Boston^10^ questionnaire was completed by the patient without the doctor´s assistance. In the case of illiterate patient, an administrative officer was appointed to assist him. When both hands were affected, the patient was asked to choose a hand that would be treated initially.

The visual analogue pain scale^11 ^(VAS) in 10 cm long, unmarked by color or symbols, ranging from no pain (zero) to severe pain (10), and was completed by patients after proper guidance.

Evaluation of sensitivity was performed on the quantitative test of cutaneous pressure threshold with a SORRI^(r)^ esthesiometer and applied by the examiner. The perception of the patient's sensitivity was estimated depending on the deformation of the monofilament due to progressive degrees of pressure resistance, being previously defined by the colors green (0,05gF / 0,49mN), blue (0,2gF / 1,96mN), violet (2gF / 19,6mN), red (4gF / 39,2mN), magenta (300 gF / 2,94N) and lack of sensitivity (no affirmative response).

The measurement of hand grip, pulp to pulp pinch, lateral pinch and tridigital pinch strength were performed using a hydraulic palmar dynamometer, adjusted to the second position and a hydraulic digital prehension dynamometer, both from Baseline(r) (Irvington, NY, USA).

To perform the measurement, the subjects were seated with their arm adducted and parallel to the trunk, elbow flexed at 90 degrees, forearm and wrist in a neutral position. Three measurements per test were performed, with the maximum strength possible, adopting the average of the values in kilogram-force.

**Figure 1.**
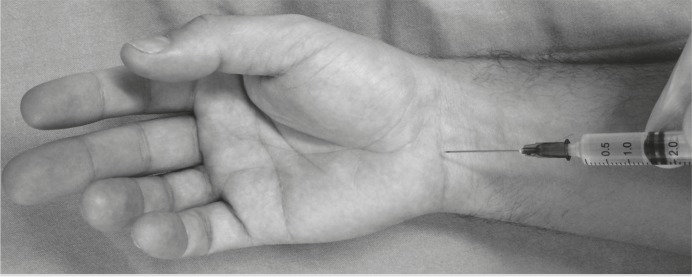
Local corticosteroids infiltration technique on carpal tunnel

Local infiltration was performed with 1 mL of methylprednisolone (40mg) and 1 mL of 2% lidocaine (20 mg), totaling 2 mL total in a single dose.^13^ The injection technique consist of inserting the needle into the proximal flexion crease of the wrist, ulnar to the *palmaris longus* tendon and tilted at 30 degrees in the horizontal plane from proximal to distal.^14^ (Figure 1)

After infiltration and serial evaluations, patients were divided into three groups according to the response to infiltration:

Group 1: No improvement. Patients that 1 month after infiltration showed no improvement of symptoms.

Group 2: Partial improvement up to 3 months. Patients who showed improvement of symptoms and recurrence in up to 3 months.

Group 3: Improvement for more than 3 months. Patients who maintained symptom relief for more than three months after infiltration.

If there was any worsening of symptoms reported by the patient during follow-up, he/she was sent to surgical release of the carpal tunnel.

Of the 100 wrists submitted to infiltration, 26 reported no improvement after the infiltration in the first post-infiltration assessment (1 month) and were referred for surgical treatment, of which 22 opted for surgery (group 1).

After 3 months of infiltration, there was recurrence of symptoms in 28 wrists, of which 20 were submitted to surgical treatment (group 2).

After 6 months, 46 wrists were in post-infiltration monitoring, of which 18 reported recurrence of symptoms and underwent surgical treatment (group 3).

**Figure 2.**
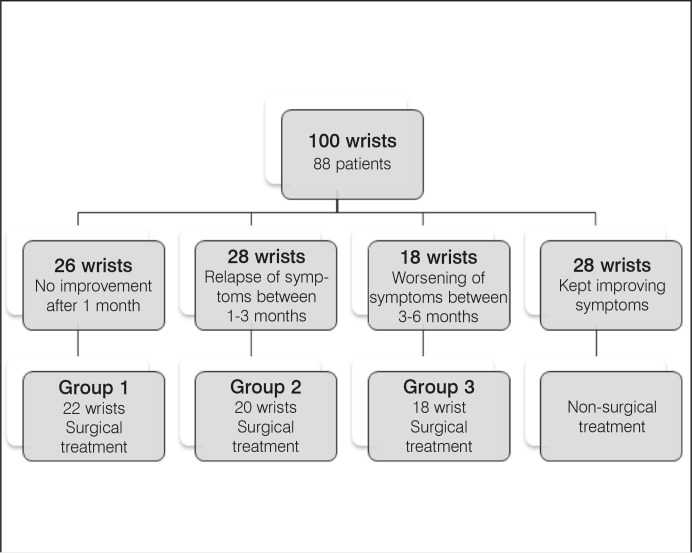
Distribution of patients according to infiltration results.

Overall, 60 wrists were submitted to surgical treatment: 22 in group 1, 20 in group 2 and 18 in group 3, as shown in Figure 2.

Patients who underwent surgical treatment were evaluated in the following periods: pre-operative, one month, three months and six months after surgery. 

### Statistical analysis

For data analysis, we used nonparametric variance analysis techniques to repeated measures.^15^ This made it possible to compare the three groups (no improvement, partial improvement and improvement for more than three months) over the four follow-up periods for surgical treatment: preoperative, one, three and six months after surgery, for the determined variables. Statistical analysis was performed using statistical software R (R Core Team, 2013).

## RESULTS

After the last post-infiltration evaluation, only 28 of the 100 wrists initially infiltrated presented no recurrence of the symptoms.

**Figure 3.**
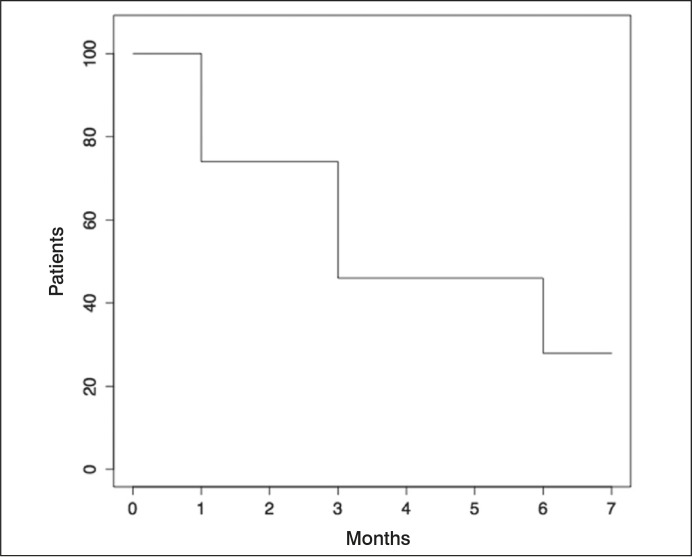
Evolution patients x time.

Figure 3 shows the deterioration of the corticosteroid effect according to the patients' follow-up time.

In group 1 (no symptom improvement after infiltration) the following average values preoperatively, one and six months after surgery, were obtained, respectively:

• VAS: 7.36; 4.5; 3.22.
• Boston questionnaire: 73.7; 41.9; 43.7.
• Force (kgf):
• Hand grip: 12.5; 9.4; 13.7.
• Pulp to pulp pinch: 2.76; 2.69; 3.72.
• Lateral pinch: 4.09; 3.86; 4.45.
• Tridigital pinch: 3.23; 2.75; 4.04.
• Sensitivity: of the 22 patients, 8 had normal sensitivity preoperatively and 15 patients had normal sensitivity six months after surgery.

In group 2 (improvement in symptoms for up to three months after infiltration), the results were as follows, in mean values in preoperative, 1 month and 6 months postoperatively:

• VAS: 8.57; 5.31; 3.56.
• Boston Questionnaire: 73.3; 46.6; 41.1.
• Force (kgf):
• Hand grip: 18.7; 10.6; 18.3.
• Pulp to pulp pinch: 3.07; 2.8; 4.4.
• Lateral pinch: 5.3; 4.0; 5.4; 5.5.
• Tridigital pinch: 4; 3.05; 4.7.
• Sensitivity: of the 20 patients, eight had normal sensitivity preoperatively and 15 patients in the normal sensitivity six months after surgery.

In group 3 (improvement of symptoms for more than three months after the infiltration), the results were as follows, in mean values in preoperative, 1 month and 6 months postoperatively:

• VAS: 6.2; 3.6; 1.4.
• Boston questionnaire: 59.2; 34.1; 27.3.
• Force (kgf):
• Hand grip: 18.4; 12.8; 17.1.
• Pulp to pulp pinch: 3.8; 3.1; 4.3.
• Lateral pinch: 5.9; 5.3; 5.59.
• Tridigital pinch: 4.6; 3.9; 4.9.
• Sensitivity: Of the 18 patients, 14 had normal sensitivity preoperatively and 16 patients with normal sensitivity six months after surgery.

There were no serious complications in any groups, both regarding infiltration and surgical treatment.

**Figure 4.**
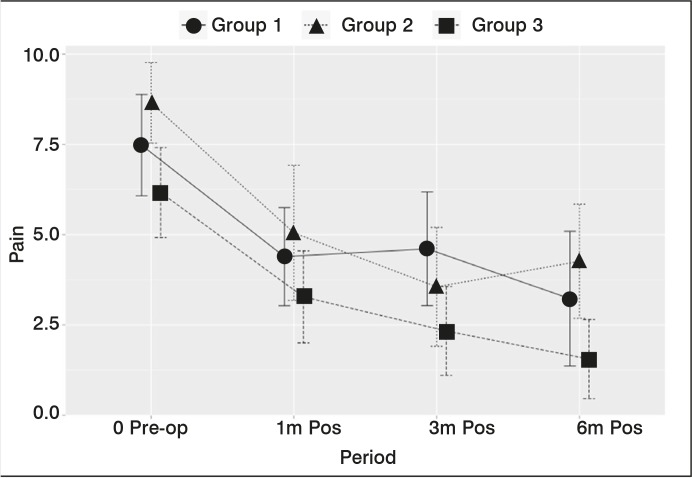
VAS: group x period.

Regarding the results on VAS, Figure 4 shows the results obtained for the three groups in postoperative follow-up and their confidence intervals.

We observed a statistically significant difference between pre and postoperative (p <0.0001), showing that surgical treatment leads to a statistically significant improvement of pain for all groups. There was also a statistically significant difference between groups with p = 0.0035, with best result of VAS for group 3.

**Figure 5.**
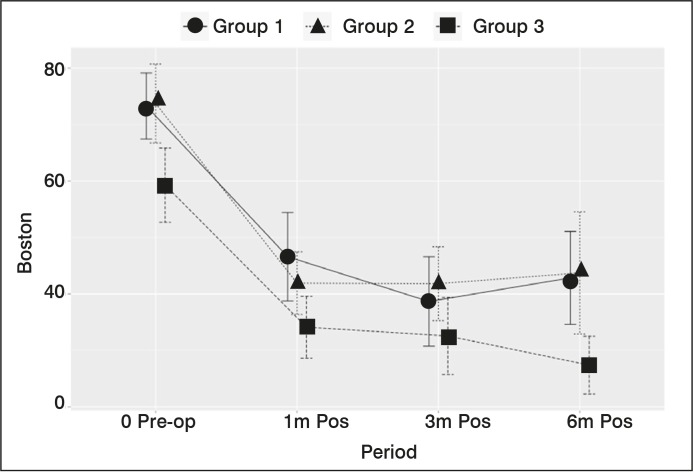
Evolution of the score on Boston-Levine questionnaire: group x period.

Regarding the results of the Boston questionnaire, Figure 5 compares periods with their confidence intervals. Group 1 is represented by the full line with a circular marker, group 2 by the dotted line with triangular marker and group 3 by the dashed line quadrangular marker. There is a statistically significant difference between groups (p = 0.0016) and periods (p <0.0001), with better results once again for group 3.

**Figure 6.**
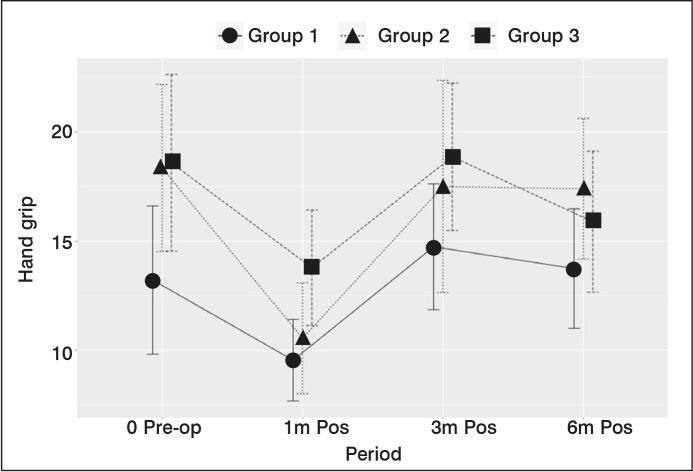
Hand grip strength x period.

**Figure 7.**
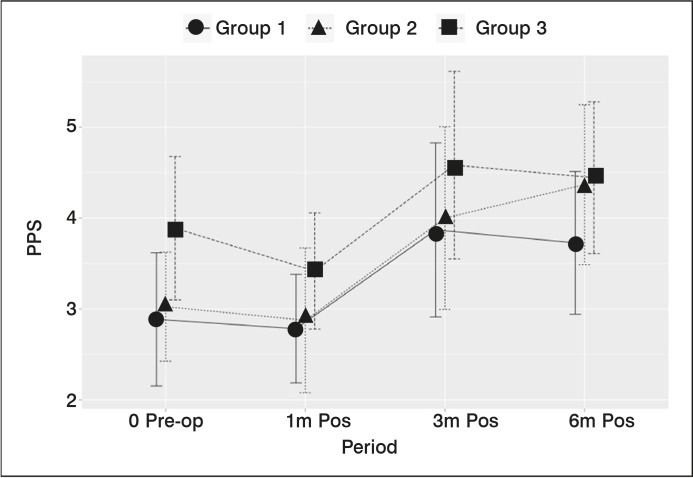
Pulp to pulp pinch strength x period.

**Figure 8.**
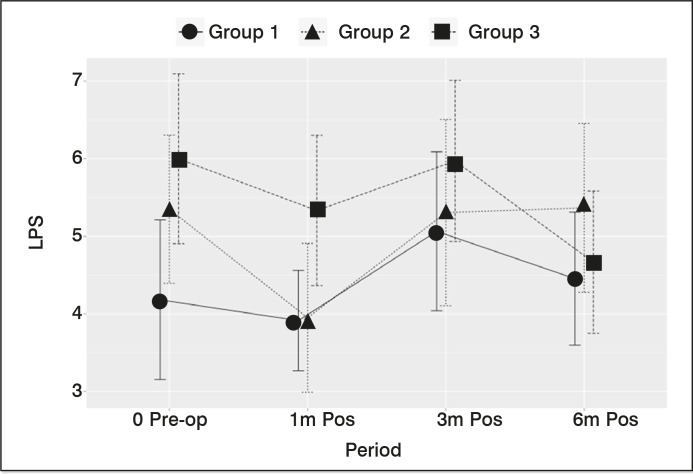
Lateral pinch strength x period.

**Figure 9.**
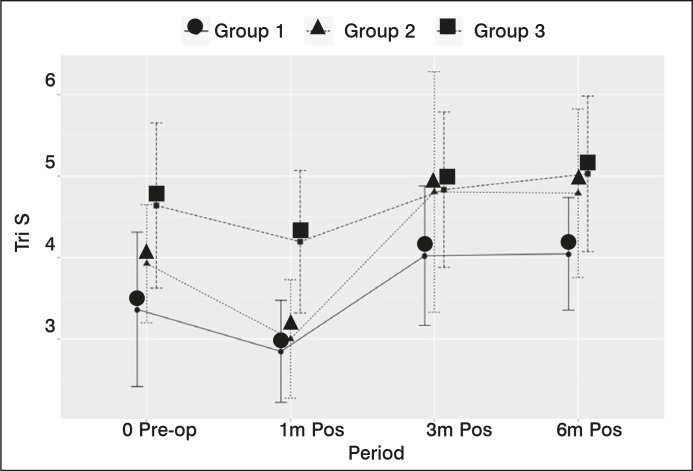
Tridigital pinch strength x period.

The results of grip strength (Figure 6) clamp pulp-pulp (Figure 7) lateral pinch (Figure 8) and tridigital pinch (Figure 9) are shown in the graphs below, with the respective confidence intervals. For all types of force analyzed, there was a statistically significant difference only between periods (p <0.0001), showing improvement in all groups postoperatively, with no statistically significant difference between groups.

To evaluate sensitivity, three categories were divided by analyzing the sensitivity in the fingertips of the second finger, autonomous area of the median nerve:

1) Normal sensitivity: green (0,05gF / 0,49mN), blue (0,2gF / 1,96mN)

2) Intermediate sensitivity: violet (2gF / 19,6mN), red (4gF / 39,2mN)

3) Poor sensitivity: magenta (300 gF / 2,94N) or absence of sensitivity.

**Figure 10.**
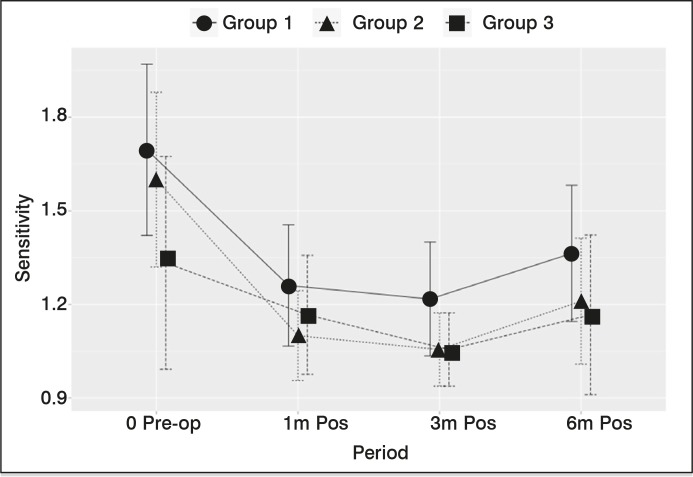
Evolution of sensitivity according to group x period

There was a statistically significant difference according to the study period, with improved sensitivity in all groups postoperatively. There was also a statistically significant difference between groups (p = 0.0281), with the group 3 showing a statistically significant result than the other, six months after surgery. (Figure 10)

## DISCUSSÃO

The local steroid injection into the carpal tunnel is a non-surgical form of treatment which provides a rate of improvement of symptoms in approximately 75% of patients. The intracanal infiltration of corticosteroids causes relief of symptoms for about a month after injection, with results shown up to two years.^16^ It shows superior to oral or systemic use of corticoide.^13^

In the present study, there was improvement in symptoms in 74 wrists (74%), a rate similar to that described by Ly-Pen *et al*.^16^ The deterioration of income over the follow-up to the progressive worsening of the patients in the assessments at three and six months after the infiltration is consistent with the description of the literature.^16^

According to Ly-Pen *et al*.,^16^ the surgical treatment is mostly indicated, especially in patients with prolonged symptoms, loss of sensation and thenar atrophy.

In our study, despite the temporary improvement with the infiltration, only 28% of the wrists had no surgical treatment. The remaining 72% did not improve or had recurrence of symptoms and, in total, 60% of fists accompanied the study underwent surgical treatment during the trial period. In the study of Meys *et al*.,^17^ the end of 12 months of follow up there was need for surgical treatment in 67.9% of patients.

Among the analyzed groups all showed a statistically significant improvement of the parameters evaluated for preoperative, showing the efficacy of surgical treatment.

Regarding the strength, it was observed in all types analyzed a decrease in the values in the first postoperative month, with a gradual increase up to six months, with a value equal to or greater than the preoperative values.^18^^,^ 19

However, regarding the visual pain scale and the score on the Boston questionnaire and sensitivity, there was a statistically significant difference between groups 1 and 3, with lower values of VAS and the Boston questionnaire and better sensitivity for group 3. These patients from group 3 were those who better responded to infiltration with relief of symptoms for a longer period, also obtaining a better outcome after surgery.

The patients from group 3 had lower pain values and better functional scores compared to patients in groups 1 and 2.

## CONCLUSION

Infiltration was shown to be a safe and effective method for temporary relief of symptoms in most patients diagnosed with carpal tunnel syndrome. There was an improvement of symptoms in all patients after surgery.

It was not possible to establish infiltration as a positive predictive factor for good results of surgical treatment, since there was an improvement in all groups postoperatively. However, we can correlate that patients in group 3, with the greatest time of symptom relief after infiltration, showed the best postoperative results.
